# Comprehensive metabolomics and transcriptomics analysis reveals protein and amino acid metabolic characteristics in liver tissue under chronic hypoxia

**DOI:** 10.1371/journal.pone.0291798

**Published:** 2023-09-25

**Authors:** Hong Liang, Kang Song

**Affiliations:** 1 Department of Basic Medical Sciences, Medical College, Qinghai University, Xining, Qinghai, China; 2 Endocrinology Department, Qinghai Provincial People’s Hospital, Xining, Qinghai, China; 3 Qinghai University Affiliated People’s Hospital, Xining, PR, China; University of California Riverside, UNITED STATES

## Abstract

At high altitudes, oxygen deprivation can cause pathophysiological changes. Liver tissue function is known to impact whole-body energy metabolism; however, how these functions are affected by chronic hypoxia remains unclear. We aimed to elucidate changing characteristics underlying the effect of chronic hypoxia on protein and amino acid metabolism in mouse livers. Mice were maintained in a hypobaric chamber simulating high altitude for 4 weeks. Livers were collected for metabolomic analysis via ultra-high performance liquid chromatography-quadrupole time-of-flight mass spectrometry. For transcriptomics analysis, we conducted RNA sequencing of hepatic tissues followed by Gene Ontology and KEGG pathway enrichment analyses. Chronic hypoxic exposure caused metabolic disorders of amino acids and their derivatives in liver tissue. We identified a number of metabolites with significantly altered profiles (including amino acids, peptides, and analogues), of which serine, phenylalanine, leucine, proline, aspartic acid, L-glutamate, creatine, 5-aminovaleric acid, L-hydroxyarginin, and g-guanidinobutyrate showed great potential as biomarkers of chronic hypoxia. A total of 2124 genes with significantly different expression levels were identified in hypoxic liver tissue, of which 1244 were upregulated and 880 were downregulated. We found pathways for protein digestion and absorption, arginine and proline metabolism, and mineral absorption related to amino acid metabolism were affected by hypoxia. Our findings surrounding the regulation of key metabolites and differentially expressed genes provide new insights into changes in protein and amino acid metabolism in the liver that result from chronic hypoxia.

## Introduction

The liver is a key hub for many physiological processes [1], including lipid and glucose homeostasis and the metabolism of macronutrients, proteins and amino acids, and biological compounds. The liver is the processing site for proteins and amino acids and as such is highly capable of metabolizing these compounds [2]. The liver is responsible for most of the protein content in the blood, the conversion of amino acids for energy production, and the processing of nitrogenous waste from protein degradation in the form of urea metabolism. Amino acid metabolism can provide energy for hepatocytes [1]. Since most amino acids, with the exception of branched chain amino acids, are absorbed efficiently by the liver, this organ can coordinate amino acid metabolism and control systemic exposure to amino acids following their uptake through the gastrointestinal tract [3]. The liver also plays a key role in integrating fuel storage and systemic metabolism [4], which includes protein and amino acid metabolism [5].

Oxygen is necessary for normal aerobic metabolism in mammals. Hypoxia is a state of deficiency in the amount of oxygen (and the resultant partial pressure of oxygen) in cells [6] and can affect many physiological processes [7]. For example,disrupted convective oxygen delivery can cause tissue hypoxia, presenting an energy challenge [8]. Hypoxia plays an important physiological role in liver function. with short-term hypoxic stress disrupting hepatic lipid metabolism [4]. Chronic intermittent hypoxia can also affect the levels of functional metabolites like tryptophan, free fatty acids, branched amino acids, and bile acids [9], whereas acute hypoxia can alter the expression of genes related to glucose metabolism in the liver [10]. However, despite the major role played by hypoxia, the mechanisms underlying its influence on hepatic protein and amino acid metabolism remain unclear.

Integrated metabolome and transcriptome analysis has been applied to the study of liver metabolic characteristics, aided by the development of bioinformatics. Such analyses allow the relationships between the expression and activity of different molecules to be established. When combined with biological functional analyses and metabolic pathway enrichment, integrated metabolome and transcriptome analysis provides a systematic and comprehensive overview of biological molecular functions and their underlying regulatory mechanisms, allowing for the identification of key metabolic pathways, genes, and metabolites. Ren et al. [11] applied this tool to the study of prostate cancer, revealing the critical metabolic pathways involved in this disease as well as a number of potential biomarkers. Similarly,. Ma et al. [12] identified ketone body and lipid metabolism disturbances related to endoplasmic reticulum stress in the liver. These findings demonstrate the potential utility of an integrated approach for investigating changes in amino acid metabolism in the liver induced by chronic hypoxia.

The objective of this study was to use metabolomic and transcriptomic approaches to conduct a comprehensive analysis of metabolic characteristics underlying chronic hypoxia in liver tissue. We developed a high-throughput screening method for liver tissue metabolites based on ultra-high performance liquid chromatography-quadrupole time-of-flight mass spectrometry (UHPLC-QTOF/MS). RNA sequencing was used to study global RNA changes in liver tissue after chronic hypoxia exposure. The relationship between differential metabolites (DMs) and differentially expressed genes (DEGs) after hypoxia exposure was clarified through network and pathway analysis. Through this approach, we were able to elucidate the characteristics underlying the changes in hepatic amino acid metabolism under chronic hypoxic conditions.

## Materials and methods

### Animals and experimental design

Six-week-old male C57BL/6J mice were purchased from SPF Biotechnology Co., Ltd. (Beijing, China). Our animal experimental protocol was approved by the Animal Protection and Use Institution Committee of Qinghai University Medical College (NO. 2022–97), China, and all animal care followed the institutional guidelines. Mice were randomly divided into either a low altitude level (LL) group (*n* = 10), maintained at an altitude of 50 m, or a high-altitude level (HL) group (*n* = 10), maintained at the equivalent of a 5000 m altitude in a DYC-300 hypobaric chamber (Guizhou Feng Lei Oxygen Chamber Co., Ltd., Guizhou, China). Both groups were exposed to their respective oxygen pressure conditions for 4 weeks in a controlled environment at 22 ± 2°C with humidity of 45–55%, and under a 12 h-12 h light-dark cycle. Water and food were available ad libitum. After 4 weeks of hypoxia or normoxia exposure, all mice were euthanized by sodium pentobarbital (Sigma-Aldrich; 50 mg/Kg) and sacrificed for the further study.

### Extraction of metabolites and metabolomic analysis

The extraction of metabolites and metabolomic analysis were performed by Shanghai Applied Protein Technology Co., Ltd. (Shanghai, China). Six mice were randomly selected from each group, and 80 mg of liver tissue was removed from each mouse (from the same location) for metabolomic analysis. Metabolites were extracted as follows: pre-cooled methanol/acetonitrile/aqueous solution (2:2:1, v/v) was added to each sample, vortexed, and then exposed to ultrasonication at −20°C for 30 min; the reaction mixture was incubated at −20°C for 10 min, centrifuged at 4°C for 20 min at 14 000 *g*, and the supernatant was collected for vacuum drying; 100 μL acetonitrile aqueous solution (acetonitrile:water = 1:1, v/v) was added during mass spectrometry, and the mixture was vortexed and centrifuged again at 4°C for 15 min at 14 000 *g* before supernatants were removed for analysis. The metabolites were identified with an Infinity 1290 UHPLC system (Agilent Technologies, Palo Alto, CA, USA) using an ACQUITY UPLC BEH Amide column (1.7 μm, 2.1 mm × 100 mm; Waters, Milford, MA, USA) coupled to a Triple TOF 6600 platform (AB Sciex, Foster City, CA, USA) set to both negative and positive ionization modes. A gradient of solvent A (25 mM ammonium acetate and 25 mM ammonium hydroxide in water) and solvent B (acetonitrile) was used for liquid chromatography separation. The flow rate was set to 0.5 mLmin^-1^, the column temperature was 25°C, and the injection volume was 2 μL. The gradient employed used 95% solvent B for 0.5 min, which was linearly reduced to 65% solvent B over 6.5 min and further reduced to 40% solvent B over 1 min, where it was maintained for 1 min until being increased again to 95% solvent B over 0.1 min, with a 3 min re-equilibration period. The electrospray ionization source conditions were: ion source gas 1 pressure at 60 psi, ion source gas 2 at 60 psi, curtain gas at 30 psi, source temperature 600°C, and an ion spray voltage floating of ± 5500 V in the positive or negative ionization modes.

Mass spectrometry raw.wiff data files were converted into the mzXML format using ProteoWizard, and XCMS software was used for peak alignment, retention time correction, and peak area extraction. The metabolites were identified using MS/MS spectroscopy to establish an internal database. Multidimensional statistical analysis of non-targeted metabolomics was performed using SIMCA-P version 14.1 (Umetrics, Umea, Sweden) and comprised principal component analysis (PCA) and orthogonal projections to latent structures-discriminant analysis (OPLS-DA). The projected importance (VIP) value of each variable in the OPLS-DA model was calculated to indicate its contribution to classification. Significance was determined using an unpaired t-test. VIP values > 1 and *p* < 0.05 indicated significance.

### Transcriptomic analysis

#### RNA extraction

Twenty milligrams tissue samples were collected from the same position in the livers of six randomly selected mice from each group for use in transcriptomic analysis (six replicates per group). Total RNA was extracted with TRIzol Reagent (Cat. no. 93289; Sigma-Aldrich, St Louis, MO, USA). The homogenate was obtained, and 250μL trichloromethane (Cat.no:10006818) was added. The solution was mixed well and left on ice for 5 min. The mixture was centrifuged at 10000 *g* and 4°C for 10 min. Then, 500 μL supernatant was extracted and the same volume of isopropyl alcohol was added, having been precooled at 4°C. The solution was mixed by inversion and left for 15 min at -20°C. After, 1 mL 75% ethanol, pre-cooled at 4°C, was added and mixed by inversion before RNA was precipitated by centrifugation at 4°C and at 10000 *g* for 5 min. The liquid was then drained and the sample left to dry for a few minutes before fully volatilizing the ethanol. The RNA was then fully dissolved by adding 100 μL RNase-Free Water. The RNA was detected with an A260/A280 absorbance ratio using a Nanodrop ND-2000 system (Thermo Scientific, Waltham, MA, USA), and the RNA integrity number was determined with a Bioanalyzer 4150 system (Agilent Technologies). Only qualified samples were used for library construction and some were used for RT-qPCR detection. The primers used for RT-qPCR assay are available in the S1 Table in S1 File.

#### Library construction and sequencing

The mRNA was purified from 1 μg of the total RNA by being annealed to oligo-(dT) magnetic beads and then subsequently fragmented in First Strand Synthesis Reaction Buffer (ABclonal Biotechnology Co., Ltd., Woburn, MA, USA). The first-strand cDNAs were synthesized using random primers and reverse transcriptase (RNase H) using mRNA fragments as templates. This was followed by the synthesis of second-strand cDNAs using DNA polymerase I, RNase H, buffer, and deoxynucleoside triphosphates. The ligated double-stranded cDNA fragments were connected to the splice sequence for polymerase chain reaction (PCR) amplification. All PCR products were purified, and the library quality was again assessed using the Bioanalyzer 4150 system. Finally, a NovaSeq 6000 (or MGISEQ-T7) sequencing platform (PE150) was used for sequencing.

#### RNA-seq data analyses

The original image data file obtained via high-throughput sequencing was converted into original sequences using CASAVA software for base recognition analysis (delivered as rawData, stored in FASTQ file format). The original data were first processed to remove joint sequences and filter out reads with low quality (where a base mass value ≤ 25 accounts for more than 60% of the whole read) and an N ratio greater than 5% (with N denoting base information that could not be determined). Clean reads were used for subsequent analysis.

Clean reads were compared to the reference genome with HISAT2 software (http://daehwankimlab.github.io/hisat2/) to map reads for subsequent analysis. The number of fragments per kilobase exon per million reads was used to estimate the level of gene expression based on gene length and number of reads. Gene Ontology (GO) and Kyoto Encyclopedia of Genes and Genomes (KEGG) pathway enrichment analyses were conducted to assess the functional enrichment of DEGs and elucidate differences observed between samples at the level of gene functional clusters. GO or KEGG functions were considered to be significantly enriched at *p* < 0.05.

### Combined metabolomics and transcriptomics analysis

A pathway map of DMs and DEGs induced by chronic hypoxia exposure was constructed based on combined metabolomics and transcriptomics analysis. R language and Cytoscape software were used for the analysis with the following criteria: VIP > 1 and *p* < 0.05 for DMs and *p* < 0.05 and | log2FoldChange | > 1.0 for DEGs. Spearman’s correlation coefficient was used to assess the correlation between DEGs and DMs.

## Results

### Effects of chronic hypoxia on liver metabolomics

**Fig 1:**
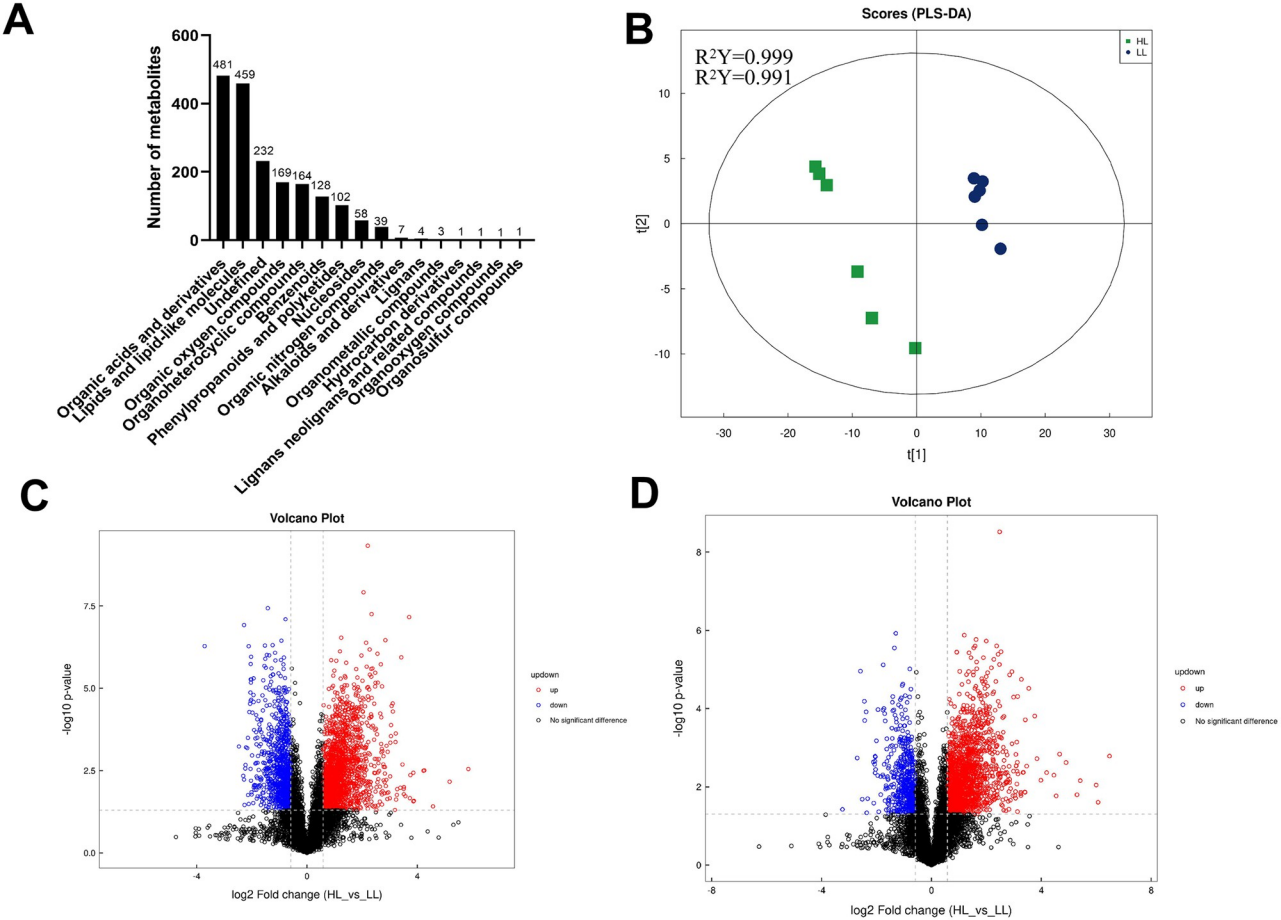
Changes in metabolic profiles of two groups mice liver. **(A)** The number of metabolites identified of two groups; **(B)** PLS-DA score plot of liver metabolites between hypoxia 4 weeks and normoxia 4 weeks. Volcano plot for high altitude group and low altitude group in **(C)** positive ion mode and **(D)** negative ion mode. Upregulated significantly differential metabolites are represented by red circles, down-regulated significantly differential metabolites are represented by blue circles, and non-significantly differential metabolites are represented by black circles.

Electrospray ionization mass spectrometry led to the identification of 1211 and 639 metabolites in the positive and negative ion modes, respectively (S1 Fig in S1 File). The subclasses and quantities of these metabolites are provided in Fig 1A. Pooled quality control samples and blank samples were used to verify the stability and reliability of the sample analysis (S2–S5 Figs in S1 File). Unsupervised multivariate analyses of metabolomics data were used to assess liver metabolic disturbances caused by hypoxic exposure. Our PCA results revealed that the LL and HL mouse groups displayed high intra-group aggregation and obvious inter-group separation, indicating that our model was reliable; these differences between the two groups were significant in both the positive and negative ion modes (S6 and S7 Figs in S1 File). Further analysis was performed using PLS-DA to better understand the variables responsible for the observed differences between the LL and HL groups (Fig 1B); the R^2^Y and R^2^Y values were 0.999 and 0.991, respectively. The differential distribution of metabolites between the LL and HL groups was visualized in volcano plots, while univariate analysis was used to assess the differences among all the metabolites detected in positive and negative ion modes with *p* < 0.05 and FC > 1.5 or FC < 0.67,respectively, being the significance thresholds. As shown in Fig 1C and 1D, 915 and 1662 metabolites were upregulated, 530 and 558 metabolites were downregulated in the positive and negative ionization modes, respectively, while the other metabolites were not significantly changed.

### Identification of candidate biomarkers for chronic hypoxia in the liver

A total of 135 and 70 significantly altered metabolites were authenticated as candidate biomarkers (VIP > 1, *p* < 0.05) in the positive and negative ion modes, respectively. In the negative ion mode, 54 metabolites were upregulated while 16 were downregulated; in the positive ion mode, 92 metabolites were upregulated while 43 metabolites were downregulated (S8 and S9 Figs in S1 File). The candidate biomarkers mainly comprised amino acids, peptides, and analogues, including serine, phenylalanine, leucine, proline, aspartic acid, L-glutamate, creatine, 5-aminovaleric acid, L-hydroxyarginine, and g-guanidinobutyrate.

### Metabolic pathway analysis of liver tissue exposed to chronic hypoxia

To explore the pathways affected by chronic hypoxia, we annotated the differential hepatic metabolites between the LL and HL groups according to pathway type in KEGG analysis. Forty-nine impacted metabolic pathways were identified. Alterations were mainly found in pathways for arginine and proline metabolism (5-aminovaleric acid, proline, creatine, L-hydroxyarginine, g-guanidinobutyrate, and L-glutamate), protein digestion and absorption (aspartic acid, leucine, L-glutamate phenylalanine, proline, and serine), and mineral absorption (leucine, phenylalanine, proline, and serine).

**Fig 2:**
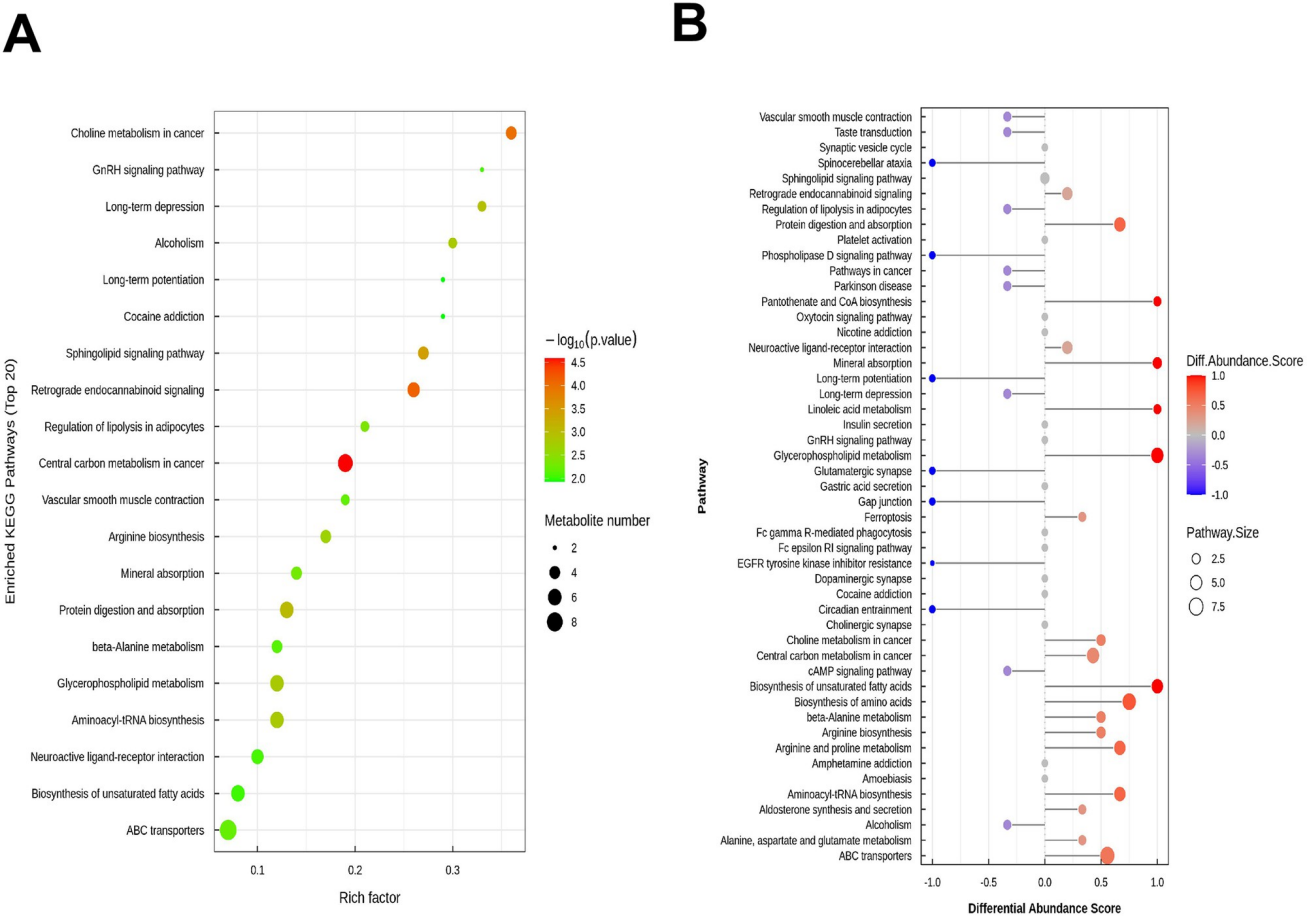
Pathways of liver tissue significantly influenced by chronic hypoxia exposure. **(A)** KEGG enrichment pathway bubble figure. Each bubble in the bubble plot represents a metabolic pathway (the top 20 with the highest significance are selected according to the p value). The abscissa where the bubble is located and the size of the bubble indicate the influence factor size of the pathway in the topological analysis. The larger the size, the larger the influence factor. The vertical axis of the bubble and the color of the bubble represent the p value of enrichment analysis (taking the negative common logarithm, that is, -log10 p-value). The darker the color, the smaller the p value, the more significant the enrichment degree. The rich factor represents the proportion of the number of differential metabolites in the pathway to the number of metabolites annotated in the pathway. **(B)** Differential abundance score map of all enriched metabolic pathways. The Y-axis represents the name of the differential pathway, and the coordinates on the X-axis represent the DA score. A score of 1 indicates that all identified metabolites in this pathway tend to be upregulated, and -1 indicates that all identified metabolites in this pathway tend to be downregulated. The length of the line segment indicates the absolute value of DA score, the size of the dot at the end of the line segment indicates the number of metabolites in the pathway, and the larger the dot indicates the more metabolites. The color of the line segments and dots is proportional to the DA score value. The darker the red is, the more inclined the overall expression of the pathway is to be up-regulated, and the darker the blue is, the more inclined the overall expression of the pathway is to be down-regulated.

The top 20 most affected pathways are shown in Fig 2A, with those significantly affected indicated by colored circles. The differential abundance score provides an average of the comprehensive variation of all metabolites in a pathway. These scores for all the significantly enriched metabolic pathways are shown in Fig 2B. Among all affected pathways, the three most affected by chronic hypoxia were those of arginine biosynthesis, protein digestion and absorption, and mineral absorption.

### Effects of chronic hypoxia on liver transcriptomics

**Fig 3:**
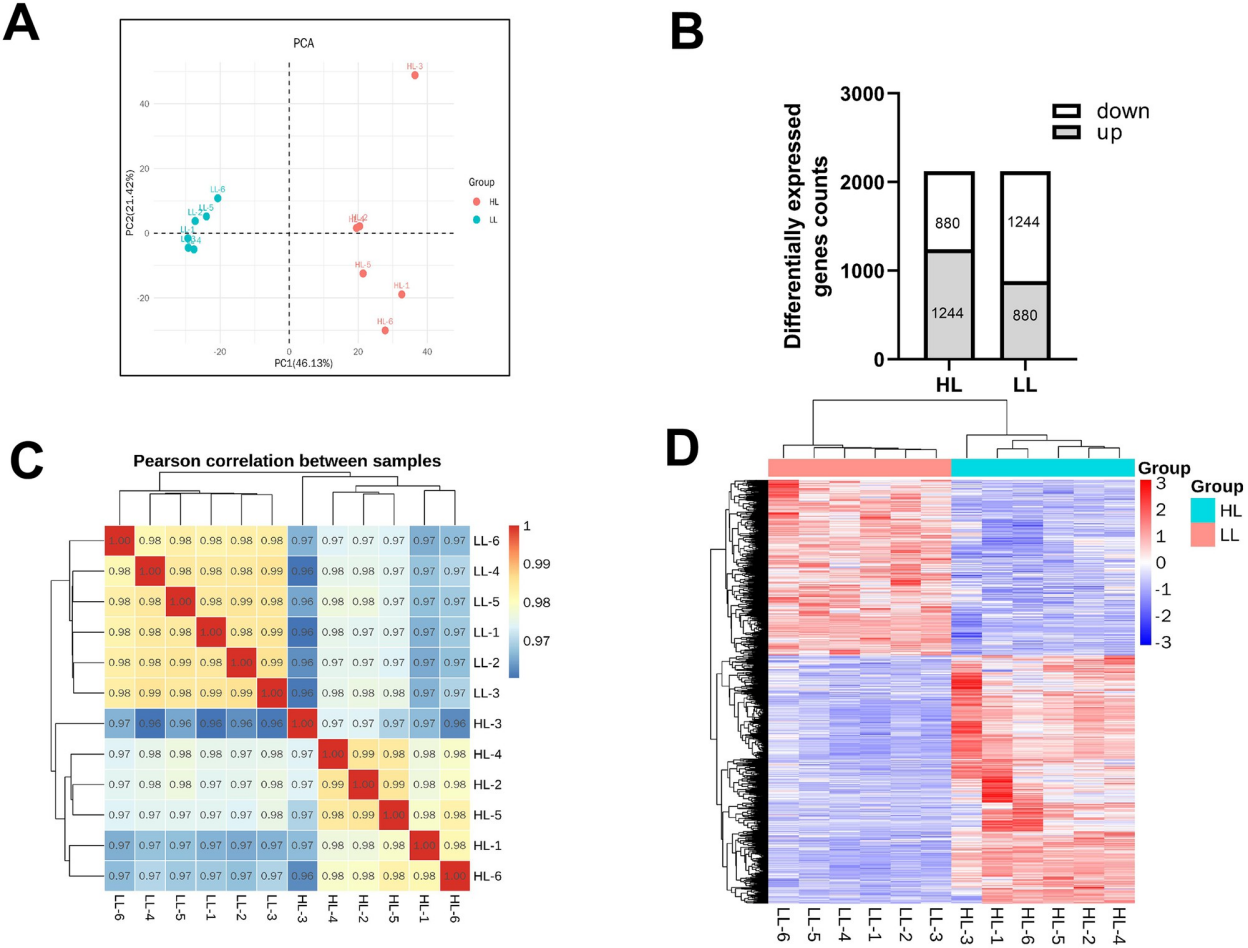
Transcriptomic analysis of liver tissues in low altitude and mice treated with hypoxia exposure. **(A)** Scores scatter plot of liver transcriptome in control mice and mice treated with hypoxia exposure; **(B)** Differential genes expression counts between low altitude group and high altitude group; **(C)** Correlation analysis of gene expression patterns in each group; **(D)** Cluster map of differential genes of two groups.

After the normalization of internal criteria, we found that the six samples of each group were closely clustered on the PCA score map (Fig 3A), indicating a significant separation between the LL and HL groups. A total of 29 121 transcripts were detected across all samples from both groups. A total of 2124 genes with significantly different expression levels were identified, of which 1244 were upregulated and 880 were downregulated in the HL group, while 880 were upregulated and 1244 were downregulated in the LL group (Fig 3B). The correlation coefficient was > 0.97, indicating that the gene expression patterns of the two groups were highly correlated (Fig 3C). Differential gene clustering was used to analyze the variation of DEGs between the HL and LL groups. Similar results were detected in the heatmap (Fig 3D).

### Effects of chronic hypoxia on gene expression

**Fig 4:**
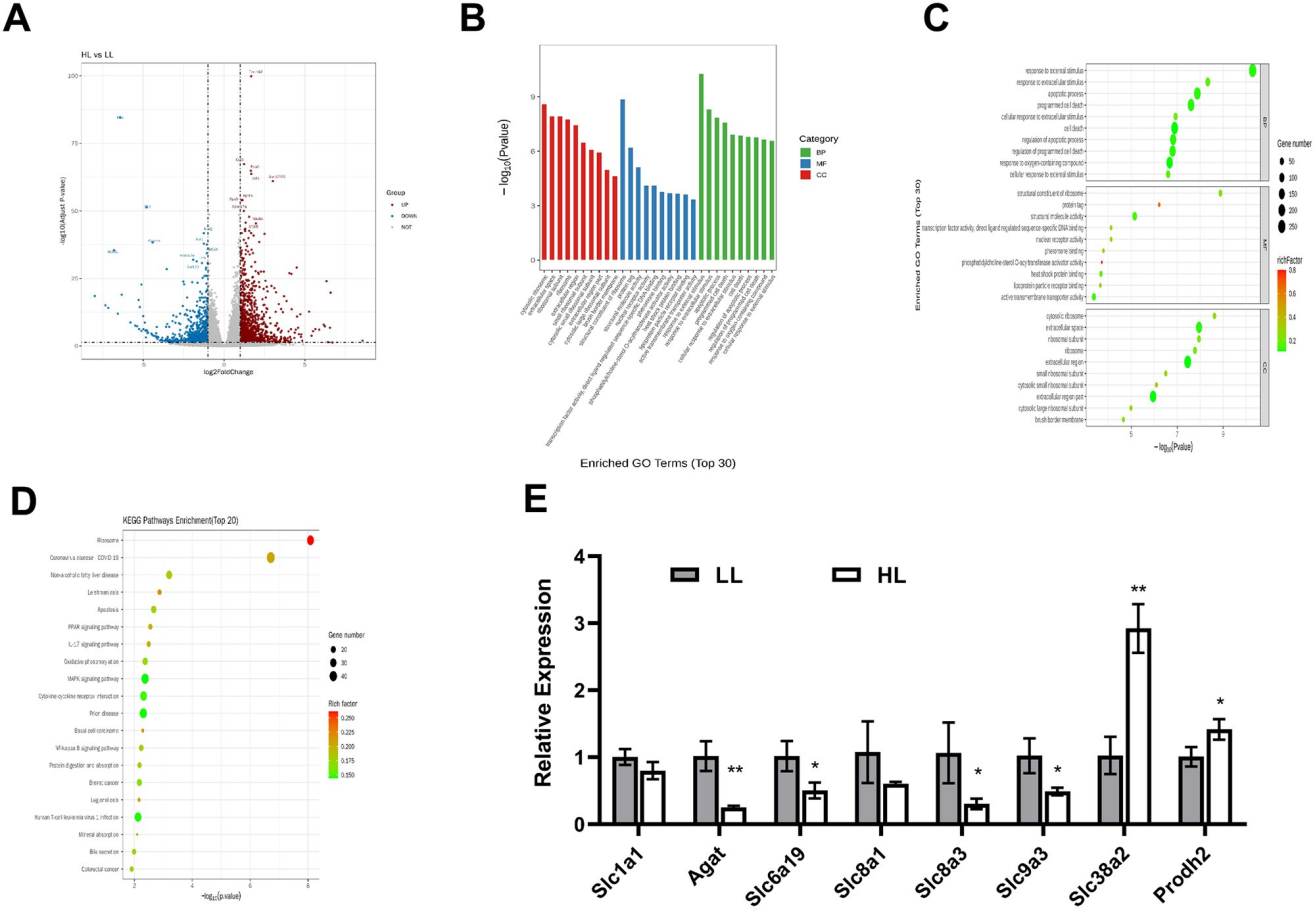
Transcriptomics profiling of the liver tissue in response to chronic hypoxia. **(A)** Volcanic plot of differential genes expression distribution. **(B)** The GO terms with the top 10 biological process (BP), cellular component (CC), and molecular function (MF). **(C)** List of GO terms with the top 30 based on the 2124 DEGs. **(D)** List of top 20 significantly enriched KEGG pathways. (E) Levels of changes in genes associated with protein and amino acid metabolism, n = 3 per group. *P < 0.05, **P < 0.01.

A volcano plot (Fig 4A) illustrated the distribution of DEGs between the control LL mice and mice from the HL group, that were exposed to chronic hypoxia. In GO analysis, the LL and HL groups were enriched for 1554 and 1309 terms, respectively. The top ten significantly enriched terms in the molecular function (MF), biological processes (BP), and cellular components (CC) categories are shown in Fig 4B and 4C. To identify the affected metabolic pathways, KEGG pathway enrichment analyses revealed that DEGs were significantly enriched in 41 pathways (*p* < 0.05), mainly those relating to protein digestion and absorption, mineral absorption, and arginine and proline metabolism (Fig 4D). RT-qPCR was used to detect the level of genes changes in protein and amino acid metabolic pathways, and the results were consistent with transcriptomics (Fig 4E).

### Analysis of DMs and DEGs under chronic hypoxia

**Fig 5:**
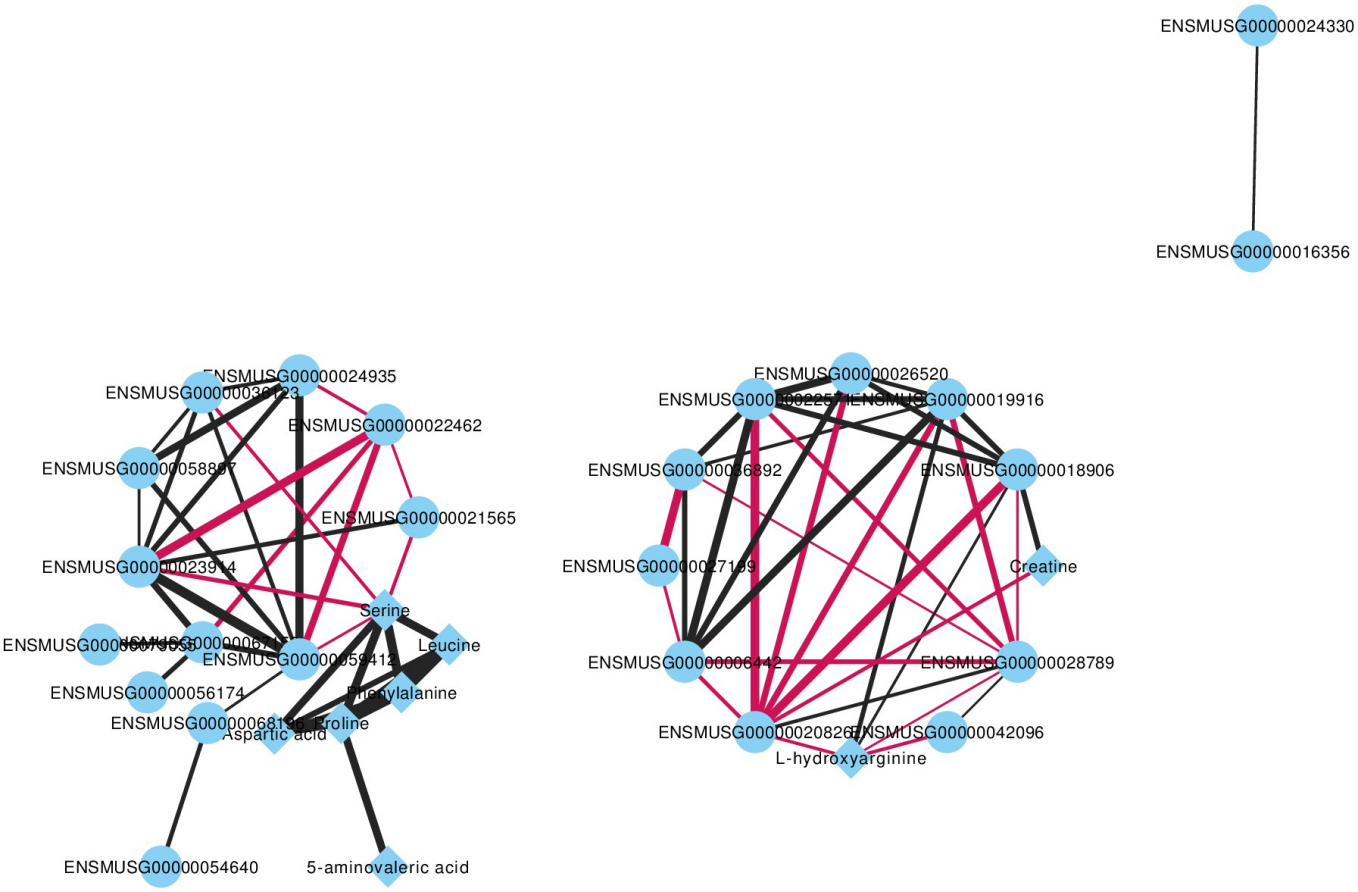
Fully connected network of differential metabolites and genes. The nodes in the rhombus indicate metabolites, and the nodes in the circle indicate genes. The color of the line represents the positive or negative value of the correlation between the two pathways (red represents negative correlation, black represents positive correlation), and the thickness of the line is proportional to the absolute value of the correlation coefficient.

Pathways involving both the DEGs and DMs identified in this study were compared, with 150 metabolic pathways being identified at the intersection of these components (S10 Fig in S1 File). The 10 pathways involving the largest number of either genes or metabolites were regarded as being affected by both gene and metabolite profiles (S11 Fig in S1 File). KEGG enrichment of DEGs and DMs indicated the enrichment of three shared pathways, namely protein digestion and absorption, mineral absorption, and arginine and proline metabolism (S12 Fig in S1 File). To screen the DEGs and DMs in the key node positions in the network, Spearman correlation network analysis was performed (Fig 5).

## Discussion

We investigated the effects of chronic hypoxia on protein and amino acid metabolism in the liver. Pathways involving protein and amino acid metabolism were identified as being affected by hypoxia in both the transcriptome and the metabolome analyses. By integrating the metabolomic and transcriptomic data, we revealed some striking alterations in multiple metabolic pathways. This provides novel insights which help us to unravel the characteristics underlying changes in hepatic protein and amino acid metabolism following exposure to hypoxia.

The body needs sufficient oxygen to maintain energetic homeostasis. Correspondingly, the liver requires energy for breaking down proteins and metabolizing the constituent amino acids [1]. Based on metabolic pathway analysis, pathways related to protein and amino acid metabolism in liver tissue are frequently altered by chronic hypoxic exposure. Non-targeted metabolomics allowed for the identification of 10 metabolites with significantly altered profiles following hypoxia, namely serine, phenylalanine, leucine, proline, aspartic acid, 5-aminovalericacid, creatine, L-hydroxyarginine, and g-guanidinobutyrate (of which all were upregulated), and L-glutamate (which was downregulated). KEGG pathway analysis further demonstrated that protein digestion and absorption, as well as arginine and proline metabolism, were upregulated due to chronic hypoxia.

The liver not only acts as a filter through its uptake of circulating amino acids but it also uses these amino acids as metabolic precursors for a large number of essential compounds, such as glucose [3]. Hypoxia is associated with multiple changes in the metabolism and energy balance of the body [13]. Leucine is an essential amino acids, and its increase under hypoxia is a mammalian response to stress [14]. Leucine, as a branched chain amino acid, can affect glucose regulation [15], and phenylalanine, serine, aspartic acid, and proline, four protein metabolites with affected profiles, are the raw materials of gluconeogenesis. Gluconeogenesis, the formation of glucose from noncarbohydrate precursors, such as amino acids or lactic acid, occurs primarily in the liver and kidney [16]. A previous study demonstrated that the hypoxic microenvironment can inhibit gluconeogenesis [17]. In this study, we found these amino acids to be at significantly higher levels under hypoxia. Some studies have found that phenylalanine accumulates in the liver under hypoxia [18], and others have suggested that low oxygen may affect the activity of dioxygenase, which promotes the metabolism of phenylalanine [19]. Further, under hypoxic conditions mice develop hypoglycemia, as evidenced by decreased pyruvate levels (despite increased gluconeogenic amino acid levels), decreased pyruvate carboxylase levels, and a decreased expression of gluconeogenic amino acid processing enzymes [20].

Glutamate is of fundamental importance to amino acid metabolism [21] and plays an important role at the cellular and system level. For instance, L-glutamate is a source of energy and is the precursor of key biological molecules in the liver. It is also an intermediate metabolite of glutaminolysis and is therefore necessary for the production of glutathione, which protects cells against oxidative stress [13]. Hypoxic conditions favor an increase in reactive oxygen species which can result in oxidative stress [22]. This study revealed that glutamate content was significant decreased in hypoxia, suggesting that the antioxidant capacity of liver tissue decreased under chronic hypoxic conditions. Cheng hang et.al., [18] showed that glutamine decreases in mouse liver as a result of hypoxia, a result that is consistent with our findings.. Serine is a non-essential amino acid supporting several processes [23], and can also be used as fuel in one-carbon metabolism [24]. Hypoxia can increase the expression of three enzymes in the serine synthesis pathway, thus increasing serine synthesis [25]. Correspondingly, our study showed an increase of serine levels in liver tissue under hypoxic exposure.

Amino acids are vital nutrients for sustaining human life [26]. They simultaneously serve as basic mediators for various intracellular and global signaling pathways. These pathways depend on the amino acid concentrations established during various input and output processes, such as those of protein digestion and absorption or those of amino acid catabolism and anabolism [27]. Amino acid transporters then modulate the cellular and circulating amino acid concentrations. In this study, the affected amino acid transporters observed were all solute carriers (SLCs), which utilize the electrochemical energy of ion gradients to drive metabolite transport across a membrane. We recorded a reduced expression of five SLCs (SLC1a1, SLC6a19, SLC8a1, SLC8a3, and SLC9a3) and an increased expression of one SLC (SLC38a2) in the liver protein digestion and absorption pathway of mice exposed to chronic hypoxia. Moreover, SLC38a2 regulates the Na^+^-dependent import of small and polar neutral amino acids [28]. It is a widely expressed sodium-dependent transporter of mainly alanine, serine, glycine, and cysteine, but can also transport glutamine, asparagine, methionine, proline, and histidine [29, 30]. Our study demonstrated an increase in three amino acids (serine, proline, and asparagine) that are transported by SLC38a2 in livers exposed to chronic hypoxia, which suggests that hypoxia activates SLC38a2.

Chronic hypoxia is a condition that is associated with an imbalance in the energy metabolism throughout the body [31]. L-arginine: glycine amidinotransferase (AGAT) is necessary for the synthesis of guanidinoacetate [32], which is converted to creatine by guanidinoacetate methyltransferase [33], mainly in the kidney and liver. Hannemann et al., [31] reported that chronic hypoxia downregulates AGAT expression in the liver and that AGAT-deficient mice are not viable under hypoxic conditions. Our transcriptomic analysis showed that AGAT gene expression was downregulated under chronic hypoxia. Creatine is synthesized in the liver and stored in the muscle. We found the level of, creatine in the liver increased under the hypoxic environment. While the body may be able to adapt to the hypoxic environment and associated metabolic changes under chronic hypoxia, other mechanisms may affect guanidinoacetate methyltransferase activity under chronic hypoxic conditions.

Proline dehydrogenase (PRODH) 2 is highly expressed genes under chronic hypoxic conditions and is involved in the arginine and proline metabolic pathway. Proline dehydrogenase/proline oxidase (PRODH/POX) is the first enzyme involved in the proline catabolic pathway, which attains critical importance when cell nutrition and oxygen are limited [34]. Liu et al., [35] found that PRODH/POX is upregulated by hypoxia both in vitro and in vivo. Adenosine monophosphate-activated protein kinase (AMPK) plays a key role in maintaining energy homeostasis [36] and is activated under hypoxic conditions [37]. PRODH/POX functions downstream of AMPK in the activation of autophagy under hypoxia in arginine and proline metabolism. We hypothesize that chronic hypoxia promotes autophagy by activating the PRODH/POX gene in the liver.

## Conclusions

By integrating transcriptomics and metabolomics analyses, we provide deeper insights into the systemic characteristics underlying liver tissue alterations due to exposure to chronic hypoxia. We demonstrate that protein and amino metabolism changes via the regulation of protein digestion and absorption and the regulation of arginine and proline metabolism. The metabolites serine, phenylalanine, proline, aspartic acid, creatine, and L-glutamate play vital roles in the hypoxic liver and have strong potential as biological markers of chronic hypoxic exposure. These results provide novel baseline references for future research on liver metabolism under hypoxic conditions.

## Supporting information

S1 File (DOCX)

S1 Data (XLS)

S2 Data (XLS)
