# Supplementary material for: Comprehensive metabolomics and transcriptomics analysis reveals protein and amino acid metabolic characteristics in liver tissue under chronic hypoxia
Source: PLoS One. 2023 Sep 25;18(9):e0291798. doi: 10.1371/journal.pone.0291798 (PMC10519603; doi:10.1371/journal.pone.0291798)
Supplement: S1 File — (DOCX) [file pone.0291798.s001.docx]

Supplementary Material

##
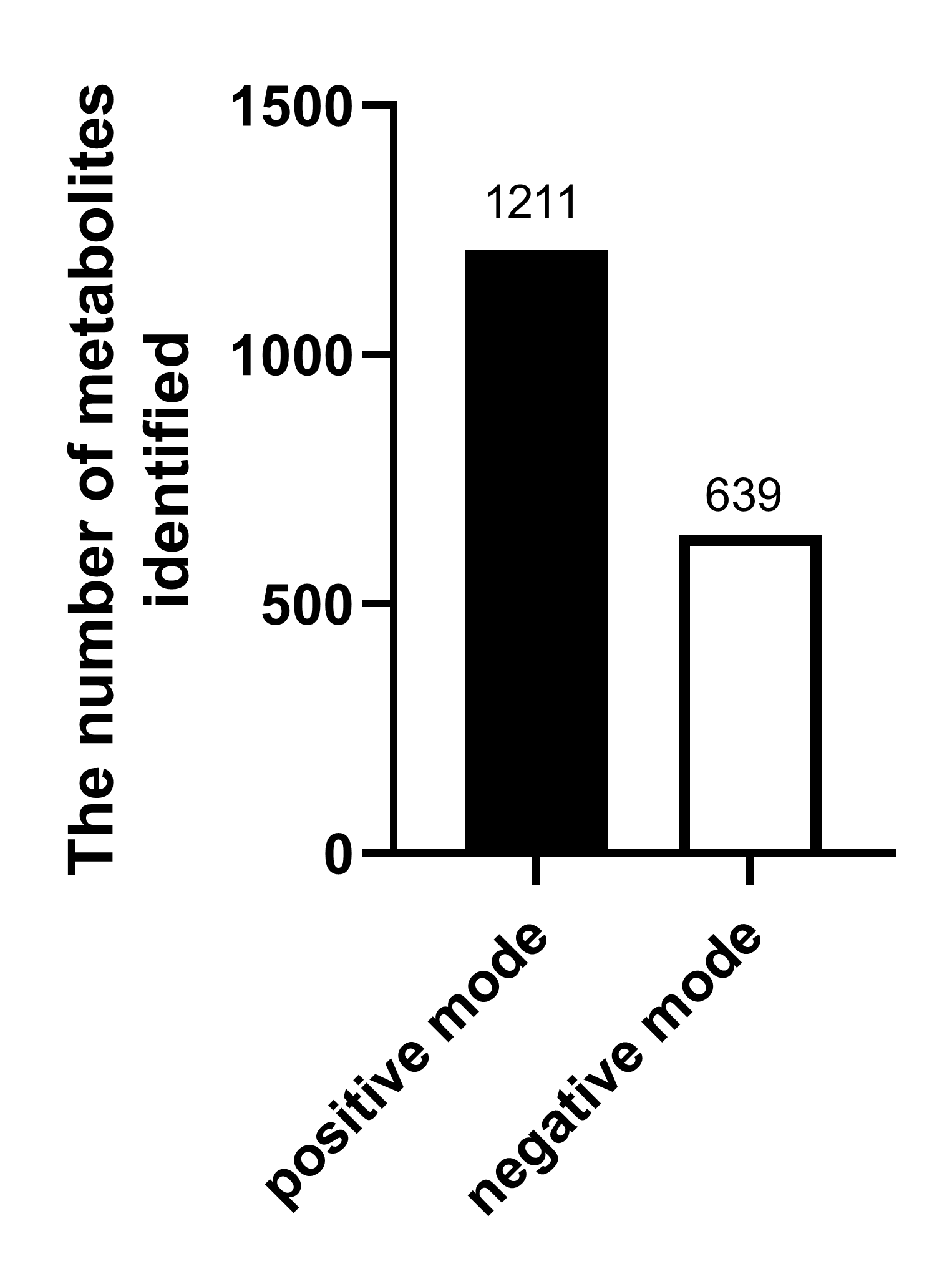


**S1 Fig1.** The number of metabolites identified by positive and negative ion models.


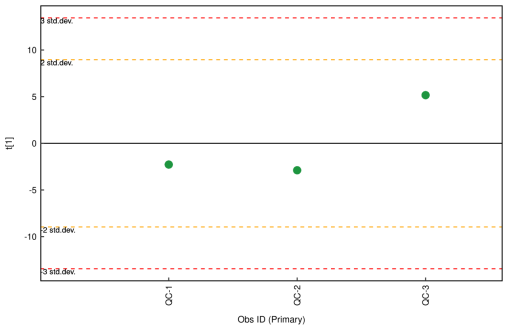


**S1 Fig 2.** Hotellings T2 plot of the population sample in positive ion mode


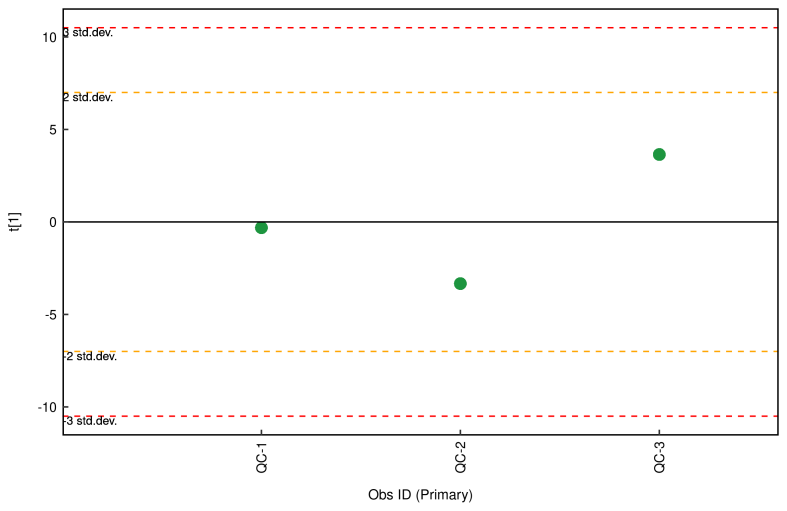


**S1 Fig 3.** Hotellings T2 plot of the population sample in negative ion mode


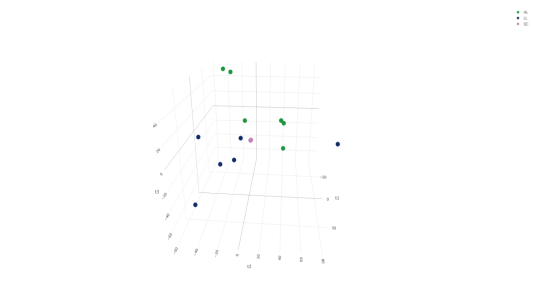


**S1 Fig 4**. PCA analysis of positive ion model population samples


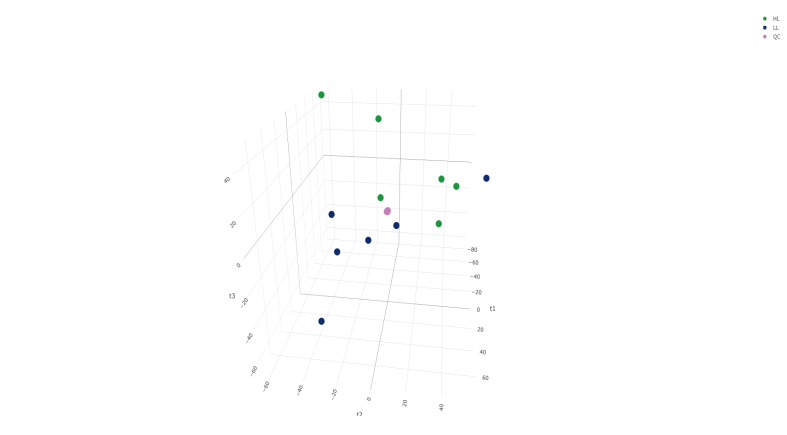


**S1 Fig 5**. PCA analysis of negative ion model population samples


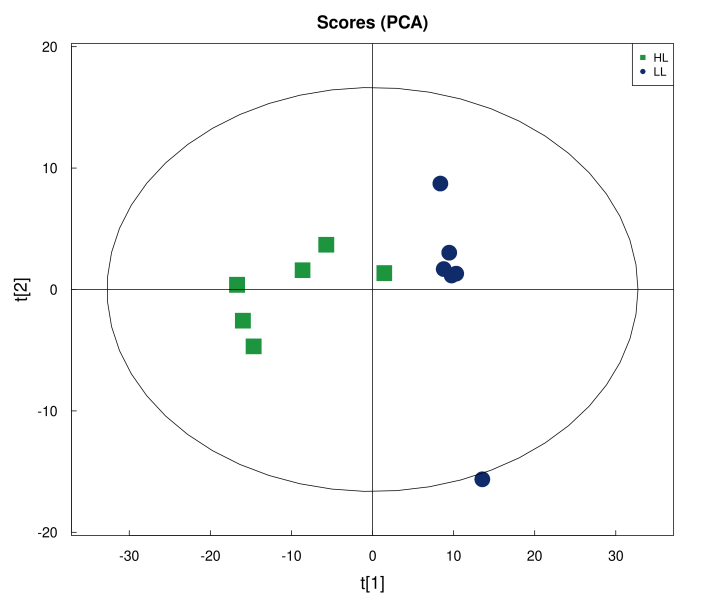


**S1 Fig 6**. PCA score map of positive ion mode


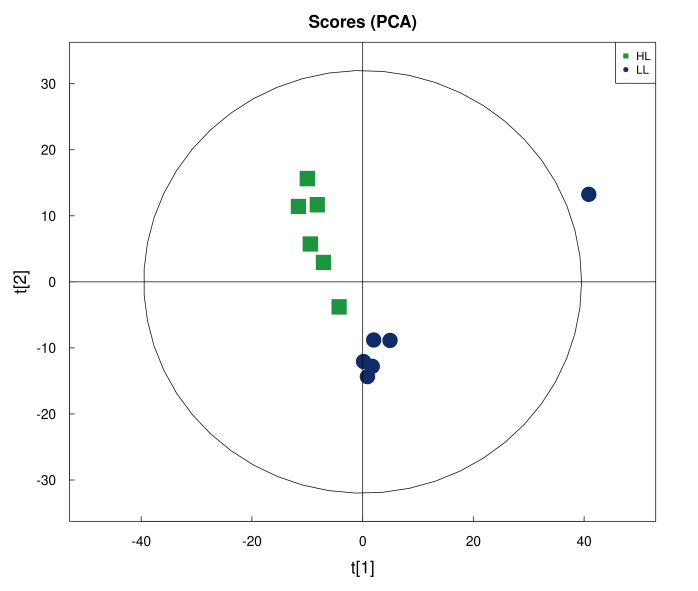


**S1 Fig 7**. PCA score map of negative ion mode


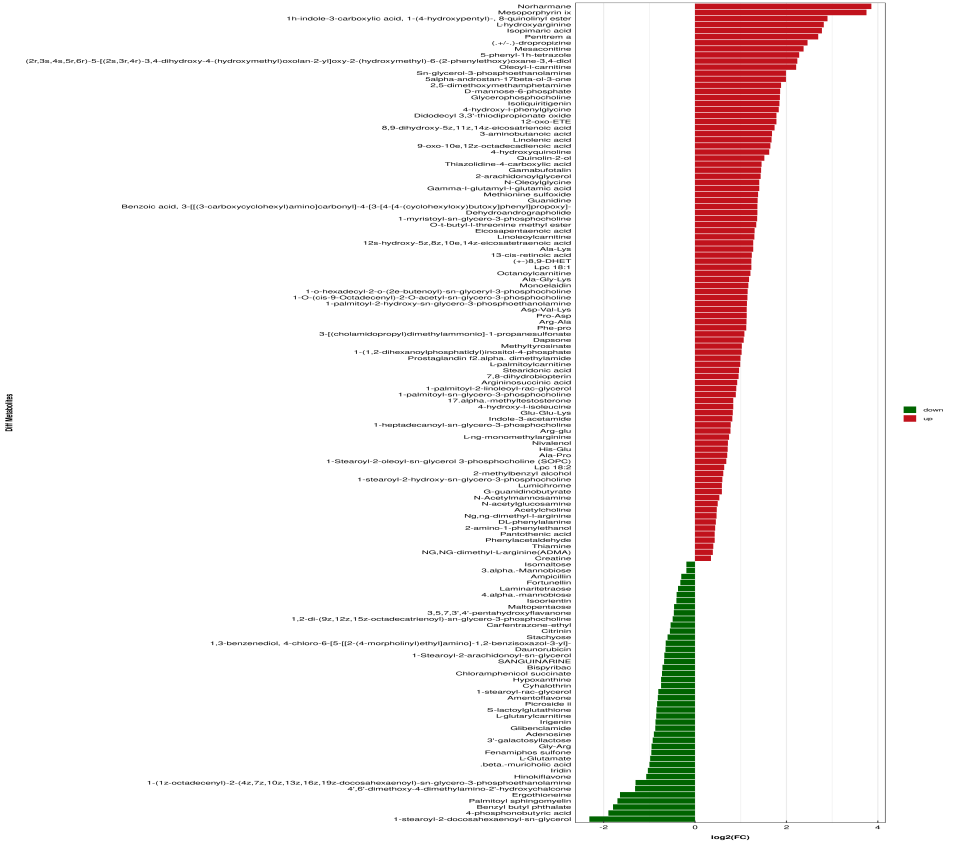


**S1 Fig 8**.Fold change analysis of significant differential metabolites expression in positive ion mode


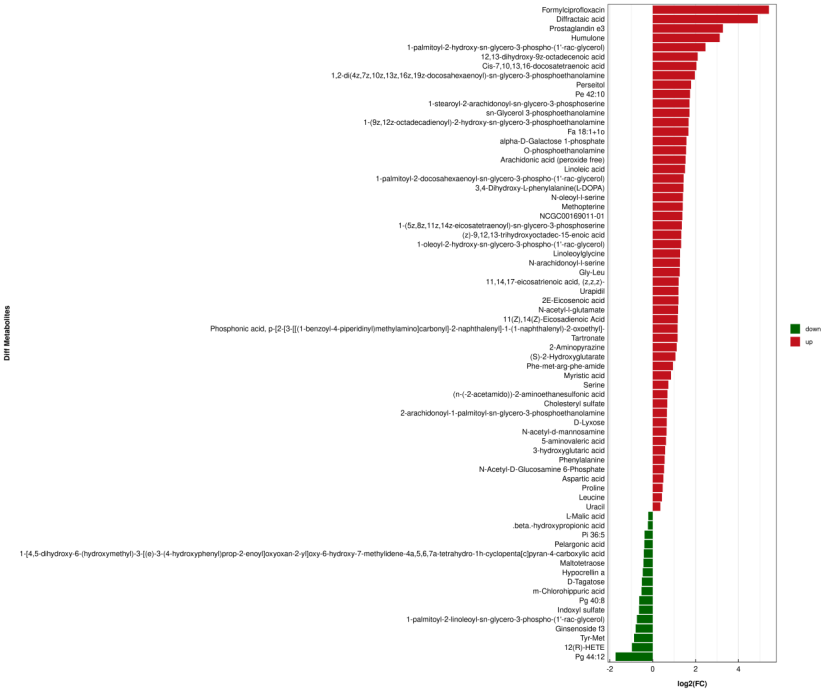


**S1 Fig 9**.Fold change analysis of significant differential metabolites expression in negative ion mode.


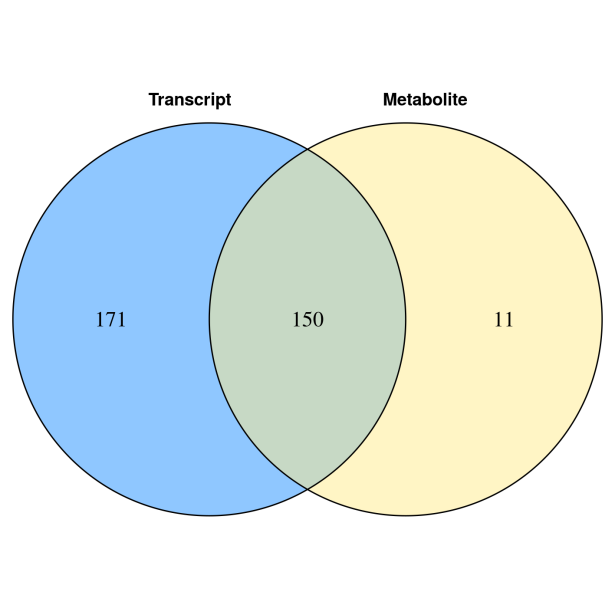


**S1 Fig 10**.Venn diagram of differential genes and differential metabolites involved in pathways


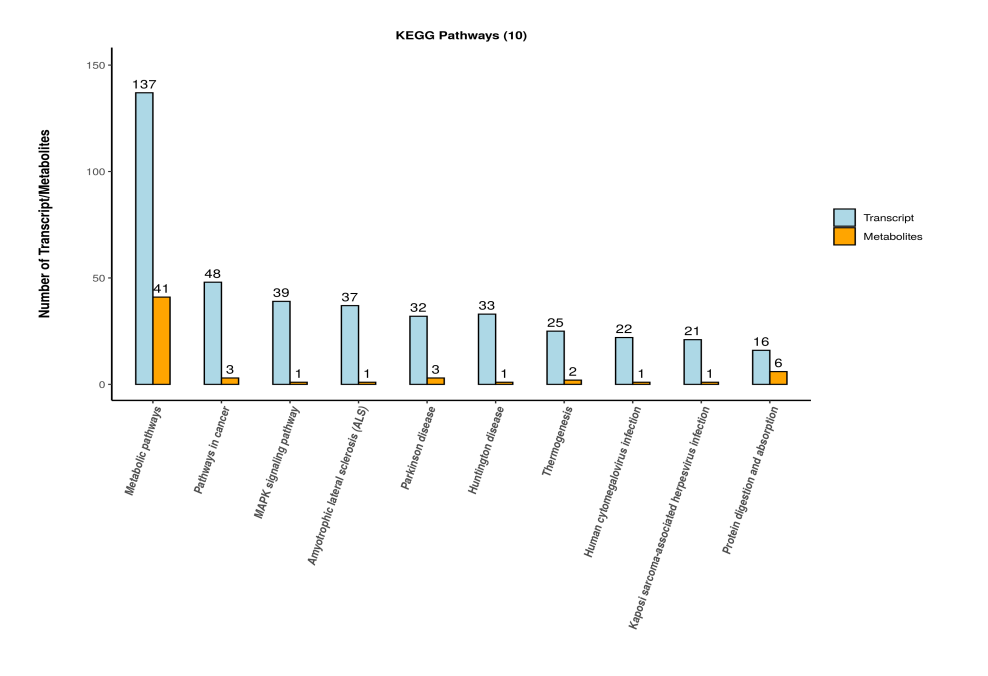


**S1 Fig 11**.The top 10 pathways with the largest number of genes and metabolites were included


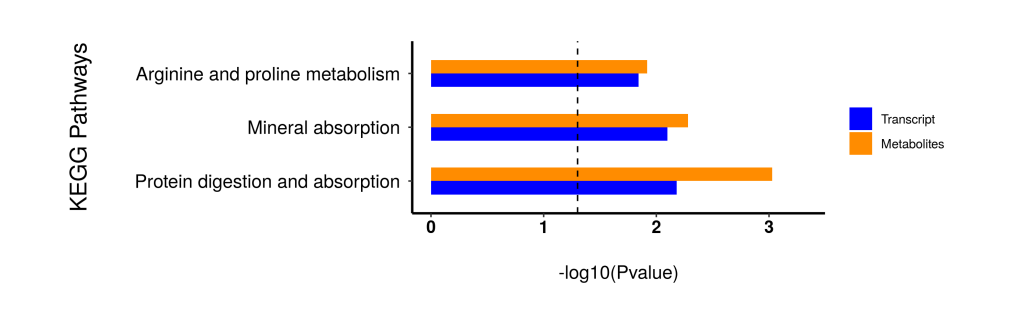


**S1 Fig 12**.KEGG enrichment of differential genes and metabolites

**S1 Table 1. Sequences of PCR primers of liver**

| Name | FORWARD(5’-3’) | REVERSE(5’-3’) | prodSize |
| --- | --- | --- | --- |
| β-actin | CTGAGAGGGAAATCGTGCGT | CCACAGGATTCCATACCCAAGA | 208 |
| Slc1a1 | CAGCGGTTCAGGGACAATACTTC | GCAGAGGATTCTAAGCCACAAGG | 83 |
| Slc9a3 | CCATGAGCTGAATTTGAAGGAT | CCAGGCATACAGCACTGACAT | 188 |
| Agat | TGAAATACTCAGTGTTGGACAGC | GAATGAATCTTGCTGGAACACAG | 275 |
| Slc38a2 | GAGTGGCATAGTGGTGATGATTGG | CGGTAGGCAGGCGGATGG | 126 |
| Slc6a19 | GGTCCTCCGCTTGCTCTCAC | CTCACCTCCTACCTTCCACTCAG | 148 |
| Slc8a1 | CGAGACTGTGTCGAACCTGAC | GATGAACATGTTAAAGGCAGCAC | 151 |
| Slc8a3 | CCTTCCTAGCCCGCCTCAC | AACCACGCCATACACAAGACTTAG | 125 |
| Prodh2 | GCCTCAGCACAGAACAGAATCAG | TCTACCAGCAGCCGCACAC | 100 |
